# Primary dedifferentiated liposarcoma of the breast: A case report

**DOI:** 10.1002/ccr3.6275

**Published:** 2022-09-05

**Authors:** Suraj Shrestha, Sanjeev Kharel, Aagon Krishna Shrestha, Ramesh Khadayat, Moushami Singh, Prafulla Shakya

**Affiliations:** ^1^ Maharajgunj Medical Campus Institute of Medicine Kathmandu Nepal; ^2^ Department of Surgery (Breast Unit) Nepal Cancer Hospital and Research Center Lalitpur Nepal; ^3^ Department of Pathology Nepal Cancer Hospital and Research Center Lalitpur Nepal

**Keywords:** dedifferentiation, liposarcoma, malignant phyllodes, primary breast liposarcoma

## Abstract

Primary breast liposarcoma is an extraordinarily rare breast malignancy. Histological subtypes including dedifferentiated are confirmed after a thorough histopathological and immunohistochemistry analysis. Liposarcoma of the breast can mimic other breast lesions. Long‐term follow‐up is needed due to the risk of local recurrence and delayed dedifferentiation.

## Graphical abstract

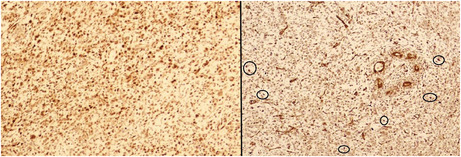

## 1 INTRODUCTION

Breast sarcoma comprises <1% of total breast malignancies and <5% of all soft tissue sarcoma. It is an extremely rare and heterogeneous group of malignancies.^1^ Liposarcoma accounts for only 0.3% of breast sarcoma. These are often misdiagnosed because of their rarity and similar clinical features mimicking other breast lesions.^2^ We report a case of a 50‐year‐old female patient who presented with a painless mass in her right breast, initially diagnosed with fibroadenoma in fine‐needle aspiration cytology (FNAC) but was diagnosed with malignant phyllodes on histopathological examination after wide local excision. The diagnosis of dedifferentiated liposarcoma was made after an immunohistochemical analysis.

## 2 CASE REPORT

A 50‐year‐old regularly menstruating P_3_L_3_ presented with a complaint of a painless right breast lump for one and half months along with gradual circumferential hyperpigmentation of the right breast for the past 30 years. There was no discharge, skin contractures, or eczema. Also, there was no history of any previous breast lesions and breast or ovarian cancer in the family.

**FIGURE 1:**
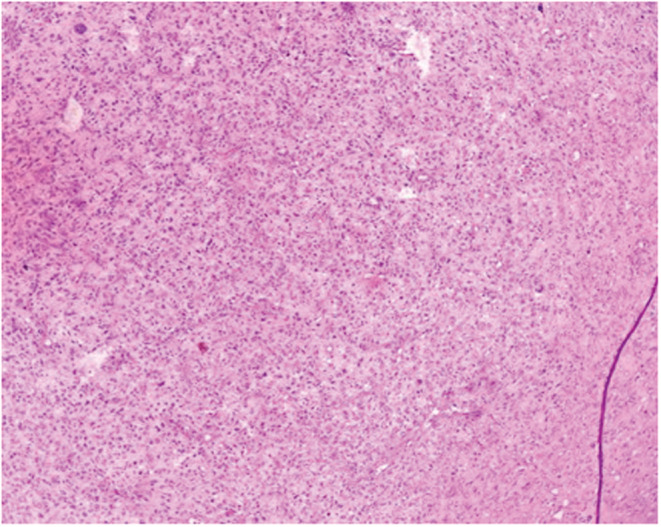
Histological section shows a cellular tumor. (H&E 100X).

**FIGURE 2:**
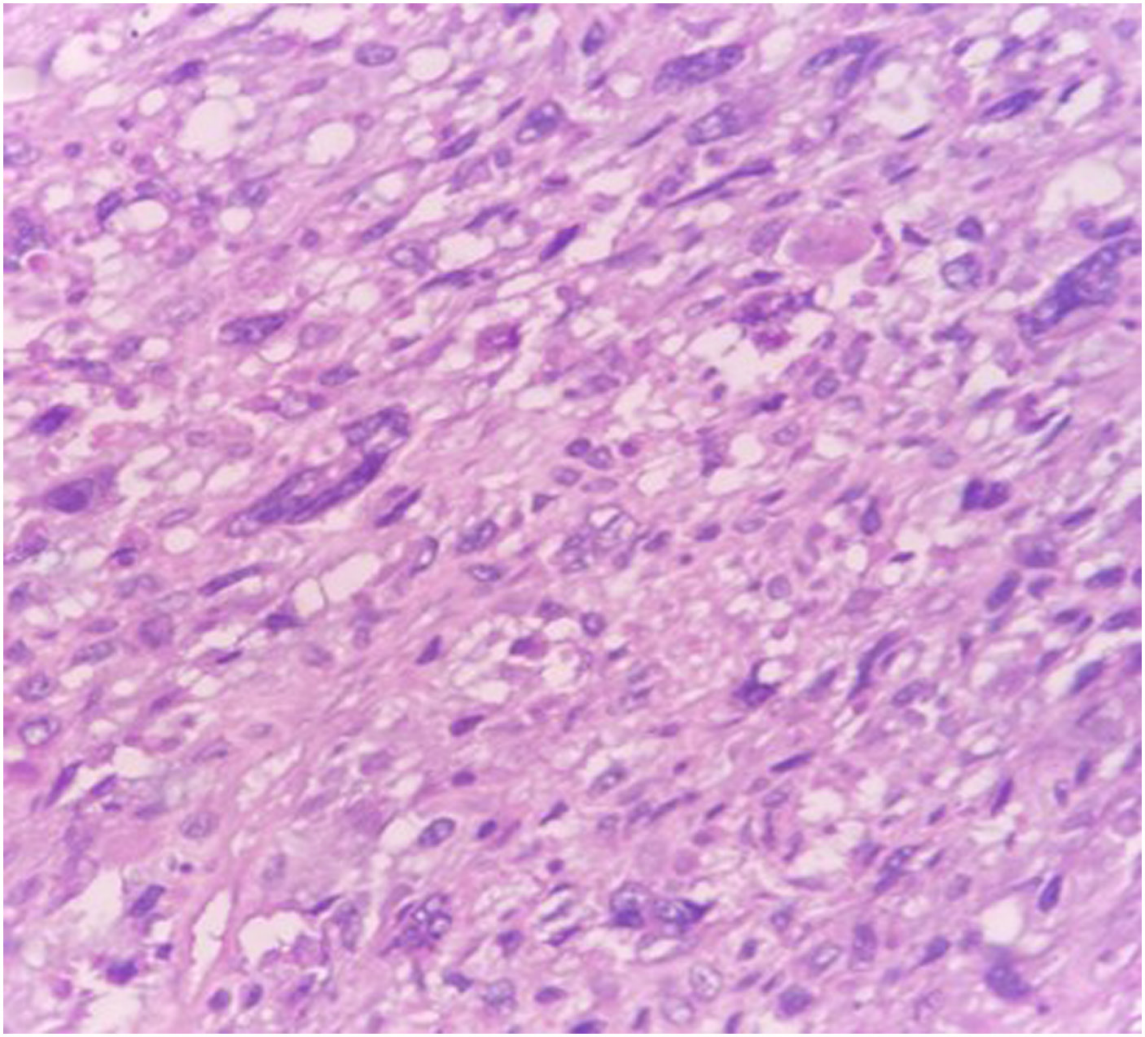
H&E section shows sheets of polygonal to spindle tumor cells exhibiting marked pleomorphism, enlarged nuclei, prominent nucleoli, and abundant pale eosinophilic to vacuolated cytoplasm.

**FIGURE 3:**
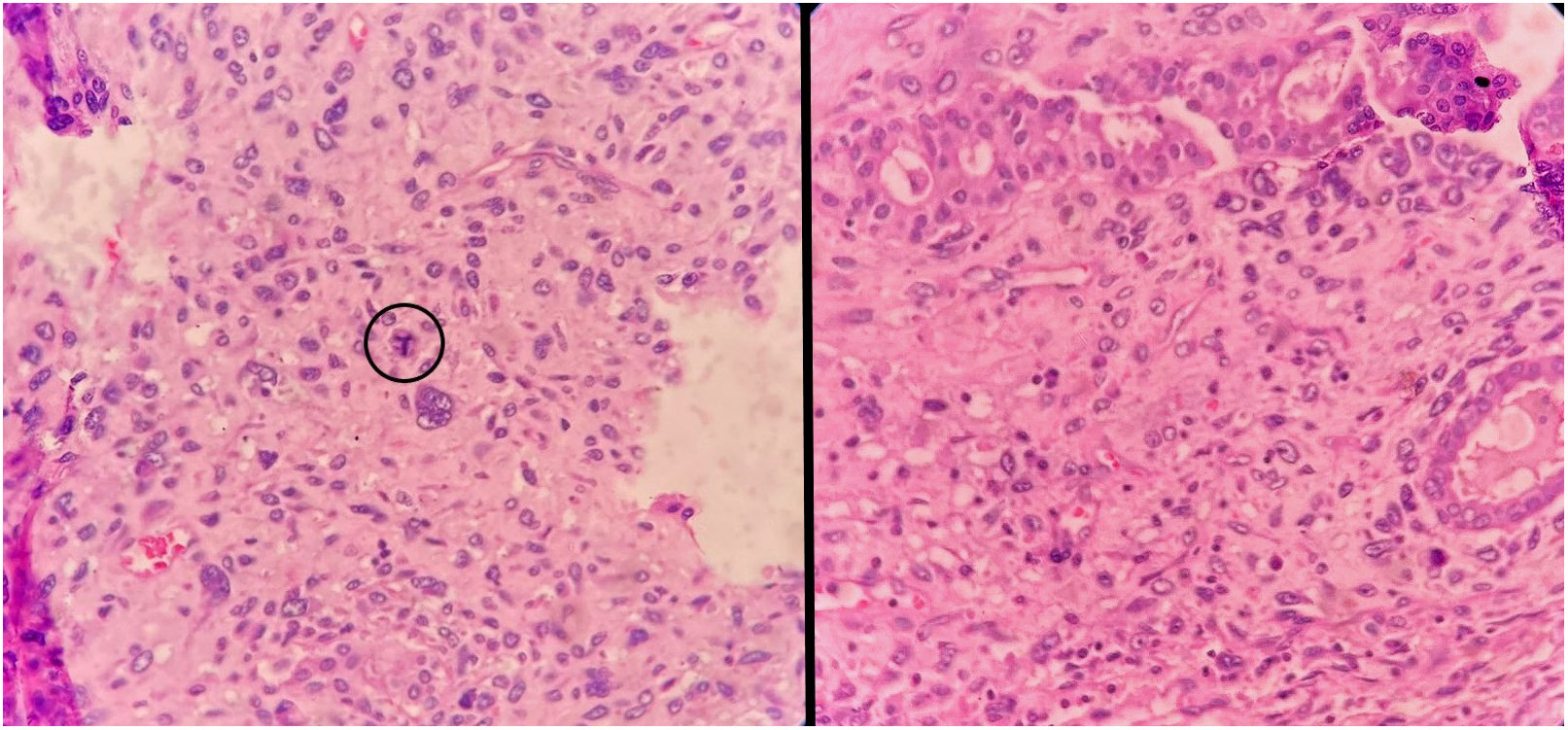
Figure shows atypical mitosis in the center (left encircled) and benign component entrapped within the tumor (right).

**FIGURE 4:**
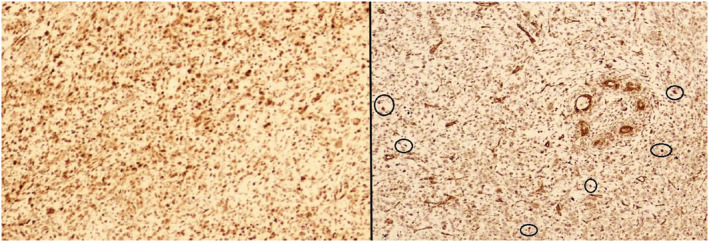
Tumor cells are positive for CDK4 (left) and MDM2 (right encircled shows nuclear positivity for MDM2).

**FIGURE 5:**
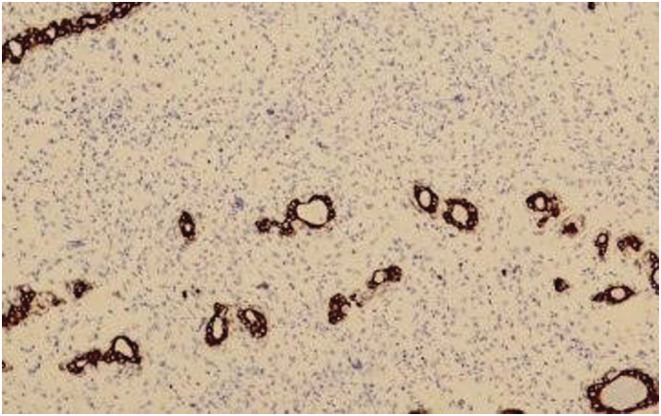
PanCK highlights the benign epithelial elements.

**FIGURE 6:**
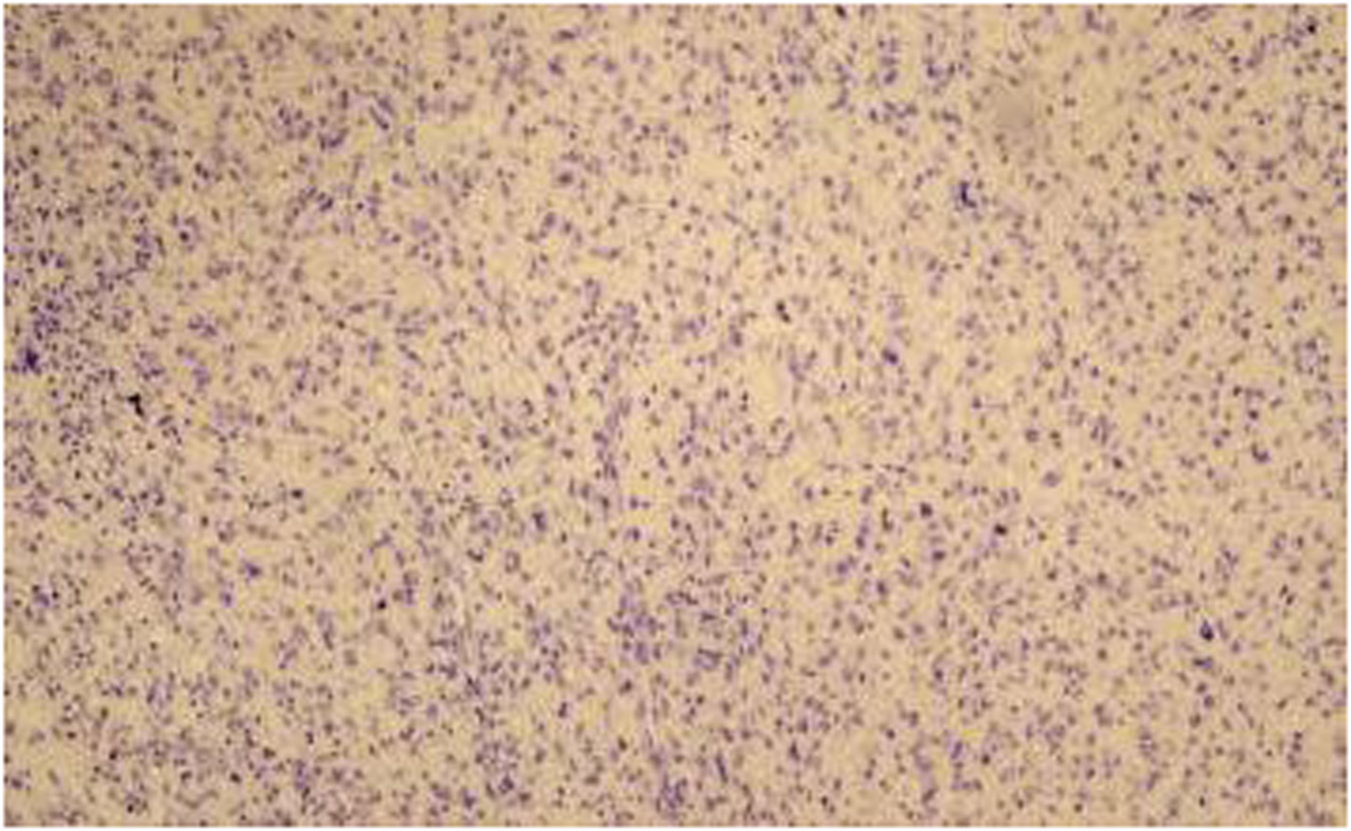
Tumor cells are negative for p63.

An ultrasonogram (USG) of the right breast revealed a well‐defined mildly hypoechoic lobulated lesion of approximately 4.3 cm × 2.4 cm × 4.1 cm at a 7 o'clock position suggestive of fibroadenoma. Contralateral breast and axillary lymph nodes were normal. FNAC of the lesion showed multiple fatty fragments consisting of varying size adipocytes without any ductal epithelial cells and atypical features, suggestive of benign breast disease. With suspicion of a giant fibroadenoma or phyllodes tumor, she underwent a wide local excision of the lump. The histopathological examination of the lump was suggestive of malignant phyllodes. However, histopathological examination of the specimen that was received at our center showed atypical oval to spindle cells exhibiting moderate to marked pleomorphism with enlarged and irregular nuclear contours. Adipocytes were noted in between the atypical cells. Mitosis was more than 10 per high power field, and myxoid areas were observed. In addition, benign ductal components were arranged in tubules and lined by luminal cuboidal to columnar cells with intact myoepithelial cells. The benign component was seen entrapped within the tumor and in the surrounding areas at the periphery of the tumor, and no leaflike pattern was noted. Also, the margins were negative for malignancy. (Figures 1, 2, 3) The immunohistochemistry shows tumor cells positive for CDK4 and MDM2. PanCK highlights the benign epithelial component of the breast and is negative for SATB2, S100, CD34, p63, SOX10, desmin, CD30, CD138, and ki67 index of 20%. All these features were favorable for dedifferentiated liposarcoma. (Figures 4, 5, 6).

With this, she was referred to our center for further management. On examination, a large horizontal incision of approximately 15 cm was present on the right breast with preserved nipple‐areolar complex and a mobile, single ipsilateral axillary lymph node was palpable. Examination of the contralateral breast and axilla was normal. Examination of all other organ systems was unremarkable.

All the blood parameters including tumor markers were within normal limits. CT scan of the chest showed postoperative changes of the right breast with diffuse skin thickening with underlying fat stranding and no definitive lesion except minimal enhancement in the retro‐areolar region along with subcentimeter axillary and supraclavicular lymph nodes without any significant abnormality in the abdomen. After these work‐ups, the patient underwent right completion mastectomy and sentinel lymph node biopsy for dedifferentiated liposarcoma of the right breast. All the sentinel lymph nodes were free of tumors. Several areas of mastectomy specimen showed areas of fibrosis, foreign body giant cell reaction along with chronic inflammation without any residual tumors.

The postoperative period was uneventful. After a tumor board discussion, the patient was planned for adjuvant radiotherapy. The patient was counseled on the prognosis and need for additional therapy but denied any.

## 3 DISCUSSION

We present an unusual case of dedifferentiated liposarcoma (DDL) of the breast. The cause for the development of liposarcoma of the breast is not well elucidated, but, it is suggested that they arise from pre‐existing benign lesions of the breast like lipoma, fibroadenoma, and phyllodes tumor.^3^, ^4^, ^5^ Ipsilateral breast radiation is also shown to have a significant association in development of breast sarcoma.^6^ Our patient had not received radiotherapy in the past nor had any benign lesion prior. They occur generally as pure primary liposarcoma or in cystosarcoma phyllodes.^7^ Mostly, the age of appearance is between 45 and 55 years. They are mostly unilateral, infiltrative, and either well‐circumscribed or multinodular. Both painful or painless soft, slowly growing masses are found irrespective of location.^8^, ^9^, ^10^

In histopathological analysis, liposarcoma appears as well‐differentiated, mixed, or dedifferentiated (high grade). The higher the grade the more is the risk of metastasis and recurrence.^11^ About 10% of cases of atypical lipomatous tumor/well‐differentiated liposarcoma (ALT/WDL) have dedifferentiation.^12^ ALT/WDL and its higher‐grade counterpart, dedifferentiated liposarcoma, are extraordinarily rare tumors in the breast. As with this case, malignant phyllodes tumor with heterogeneous liposarcomatous differentiation and high‐grade metaplastic breast carcinoma are the two major differential diagnoses for primary breast DDL.^13^ Fine‐needle aspiration (FNA) biopsy is a widely accepted diagnostic procedure for evaluating epithelial breast lesions because of its cost‐effectiveness and high sensitivity and specificity. However, for diagnosing different sarcoma, the sensitivity and specificity of FNA is still low. Therefore, a core or excisional biopsy is opted for diagnosing breast sarcomas.^1^, ^14^ Nevertheless, recognizing such lesions can avoid unnecessary sentinel and axillary lymph node dissection. USG and FNAC were performed in our case; the findings of both were mimicking a benign breast lesion. Immunohistochemistry tests for desmin, vimentin, α‐SMA, CK, leukocyte common antigen (LCA), CD34, human melanoma black 45 (HMB45), epithelial membrane antigen (EMA), and S100 are performed in sarcomas to differentiate other tumor types and make a final diagnosis.^15^ Similarly, an extensive histopathological examination and immunohistochemical examination were done in our case for reaching a diagnosis.

The procedure recommended for the curation of liposarcoma is complete resection with negative margins (R0), but still, the debate remains on the use of either breast conservative surgery (BCS) or mastectomy. A 20 cases’ review showed that axillary lymph node dissection is usually not necessary as the axilla is not a frequent metastasis region,^8^ while better survival outcomes were seen with breast‐conserving surgery than mastectomy in M0 patients.^1^ Excision of the tumor is done in well‐differentiated/dedifferentiated subtype, which is less aggressive. With multiple recurrences, highly malignant dedifferentiated histology with significant metastatic potential can be seen.^16^

The significant prognostic factors are tumor size, grade, histology, and radiation history. A significantly worse prognosis and high rate of recurrence are seen in primary breast sarcoma.^1^ The five‐year survival rate is approximately 50% in liposarcoma of the breast.^17^ Though the use of radiotherapy and chemotherapy in non‐metastatic primary breast sarcoma is not clear, high‐risk patients should be counseled for use of adjuvant chemotherapy and radiation therapy including difficulty in obtaining negative surgical resection status, higher‐grade liposarcoma, or size >5 cm.^16^, ^18^ There are no clear guidelines for the surgical treatment and any subsequent specific therapy because of its rarity and poor response to therapies.^19^ In these cases, there is a risk of local recurrence or delayed dedifferentiation so long‐term follow‐up is needed.^20^ Our patient is on‐regular follow‐up even though she denied adjuvant therapy and is doing well till one year of surgery.

## 4 CONCLUSION

Liposarcoma of the breast is difficult to diagnose with imaging studies, and biopsies can be misleading. Immunohistochemistry analysis can help in a definitive diagnosis. Long‐term follow‐up is needed because of the risk of local recurrence or delayed dedifferentiation.

## AUTHOR CONTRIBUTIONS

Prafulla Shakya involved in conceptualization, resources, writing–original draft, and writing–review and editing. Aagon Krishna Shrestha, Ramesh Khadayat, and Sanjeev Kharel involved in conceptualization, investigation, and writing–review and editing. Suraj Shrestha involved in conceptualization, supervision, and writing–review and editing. Moushami Singh involved in investigation, resources, and writing–review and editing. Prafulla Shakya = Senior author and manuscript reviewer. The manuscript is reviewed and approved by all the authors.

## CONFLICT OF INTEREST

None to declare.

## CONSENT

Written informed consent was obtained from the patient herself and her daughter for the publication of this case report. A copy of the written consent is available for review by the editor in chief of this journal on request.

## Data Availability

All the necessary data and materials are within the manuscript.
